# Reduction of HIV-associated excess mortality by antiretroviral treatment among tuberculosis patients in Kenya

**DOI:** 10.1371/journal.pone.0188235

**Published:** 2017-11-16

**Authors:** Dickens O. Onyango, Courtney M. Yuen, Kevin P. Cain, Faith Ngari, Enos O. Masini, Martien W. Borgdorff

**Affiliations:** 1 Kisumu County Department of Health, Kisumu, Kenya; 2 Harvard Medical School, Boston, Massachusetts, United States of America; 3 U.S. Centers for Disease Control and Prevention – Kenya, Kisumu, Kenya; 4 National Tuberculosis, Leprosy and Lung Disease Program, Nairobi, Kenya; University of Ottawa, CANADA

## Abstract

**Background:**

Mortality from TB continues to be a global public health challenge. TB ranks alongside Human Immunodeficiency Virus (HIV) as the leading infectious causes of death globally. HIV is a major driver of TB related morbidity and mortality while TB is the leading cause of mortality among people living with HIV/AIDS. We sought to determine excess mortality associated with HIV and the effect of antiretroviral therapy on reducing mortality among tuberculosis patients in Kenya.

**Methods:**

We conducted a retrospective analysis of Kenya national tuberculosis program data of patients enrolled from 2013 through 2014. We used direct standardization to obtain standardized mortality ratios for tuberculosis patients compared with the general population. We calculated the population attributable fraction of tuberculosis deaths due to HIV based on the standardized mortality ratio for deaths among TB patients with HIV compared to TB patients without HIV. We used Cox proportional hazards regression for assessing risk factors for mortality.

**Results:**

Of 162,014 patients included in the analysis, 6% died. Mortality was 10.6 (95% CI: 10.4–10.8) times higher among TB patients than the general population; 42% of deaths were attributable to HIV infection. Patients with HIV who were not receiving ART had an over four-fold risk of death compared to patients without HIV (aHR = 4.2, 95% CI 3.9–4.6). In contrast, patients with HIV who were receiving ART had only 2.6 times the risk of death (aHR = 2.6, 95% CI 2.5–2.7).

**Conclusion:**

HIV was a significant contributor to TB-associated deaths in Kenya. Mortality among HIV-infected individuals was higher among those not on ART than those on ART. Early initiation of ART among HIV infected people (a “test and treat” approach) should further reduce TB-associated deaths.

## Introduction

Tuberculosis (TB) is an infectious disease caused by infection with Mycobacterium tuberculosis. Mortality from TB continues to be a global public health challenge. TB ranks alongside Human Immunodeficiency Virus (HIV) as the leading infectious causes of death globally [1]. In 2015, of the 10.4 million people who developed TB, 1.8 million died, of whom 0.4 million were persons infected with HIV [2]. The African region has the highest rates of TB incidence and mortality, while Kenya is one of the 22 countries with the highest TB burdens [3]. TB notifications in the country increased more than six fold from 1990 (50 cases per 100,000) to a peak of 319 per 100,000 in 2006, largely due to the HIV epidemic; TB notifications declined from 2007 onwards but have remained persistently high TB among HIV-positive individuals (over 1,800 cases/100,000 population) [4, 5]. HIV prevalence among adults aged 15 to 64 years in the country was 5.6% in 2012 [6].

The high HIV prevalence in Kenya could be expected to lead to high mortality among TB patients who are co-infected with HIV. HIV is a major driver of TB related mortality while TB is the leading cause of mortality among people living with HIV/AIDS [7, 8]. Major factors associated with mortality among TB/HIV co-infected people include delay in diagnosis of either disease [9] and delay in initiating antiretroviral therapy (ART)[10]. Early initiation of ART in TB patients has been proven to improve survival of those who are co-infected [11–13].

Other risk factors for mortality have been described by numerous studies, which have consistently identified incomplete treatment due to default, drug resistance, male gender, and immunosuppression as predictors of mortality [14–19]. However, other risk factors have been identified only in certain settings, including having TB due to recent transmission [17] or having less than six years of formal education [17]. There have been conflicting reports on which age group is associated with mortality: some studies have reported higher risk among younger age groups [15, 16] while others have reported higher risk among older age groups [19, 20]. A study from western Kenya reported having unknown HIV status and not being on ART during TB treatment as risk factors [21].

Knowledge is lacking on the impact of HIV on mortality among TB patients in Kenya and the impact that ART has had on TB/HIV-associated mortality in a programmatic setting. This study sought to determine HIV-associated excess mortality, estimate the current impact of ART and the potential effect of further ART expansion on HIV-associated mortality in TB patients, and identify risk factors for mortality during TB treatment in Kenya, a middle-income country with high burdens of both TB and HIV.

## Methods

### Setting

In Kenya, TB is diagnosed and treated according to the national treatment guidelines adopted from World Health Organization recommendations [22, 23]. Tuberculin skin test, chest radiography and sputum smear examination are done to support clinical evaluation. More recently, the Gene Xpert MTB/Rif Assay has been introduced, and its use is being scaled up. New TB patients go through a four-drug (rifampicin, isoniazid, pyrazinamide and ethambutol) intensive phase of treatment for two months and a continuation phase of four months (isoniazid and rifampicin). Patients who have previously been treated for TB (i.e., retreatment patients) are put on an intensive phase of three months (rifampicin, isoniazid, pyrazinamide and ethambutol) and five months continuation (isoniazid and rifampicin). Patients with certain types of extra-pulmonary disease specifically tuberculous meningitis or bone and joint disease are treated for 12 months; two months of intensive phase and ten months continuation.

### Data source

This study used data from the electronic TB surveillance system, Tuberculosis Information from Basic Unit (TIBU), which was introduced in Kenya in 2012. All public, faith-based, and private treatment centers in the country enter individual-level data into this centrally located system. Data used in this analysis were drawn from 3511 health facilities. An assessment conducted in one district and clinic reported that nearly 100% of all treated TB patients were captured in the TIBU system [24]. TIBU variables included in the risk factor analysis were treatment outcome, age, sex, region, HIV status, ART use, smear results, disease type, whether or not Directly Observed Therapy (DOT) was done, the person administering DOT, HIV status, ART status of HIV-infected patients, and previous TB treatment history.

### Statistical analysis

We included all patients in the electronic register who had started their treatment during 1^st^ January 2013 through 31^st^ December 2014. Children aged less than 15 years were excluded from this analysis due to the uncertainty of a TB diagnosis in children and under-reporting of cases [21, 25, 26]. Before starting the analysis we performed range and consistency checks.

Excess mortality by age and sex was determined by comparing the expected number of deaths (calculated using age- and sex-specific mortality rates published by the Kenya National Bureau of Statistics [27]) with the observed number of deaths in this treatment cohort. We calculated the population attributable fraction (PAF) of mortality among TB patients attributable to HIV by calculating a standardized mortality ratio (SMR) for HIV-infected compared with HIV-non-infected TB patients. PAF (%) was calculated as: (SMR-1)/SMR*100.

To determine risk factors for mortality, we conducted bivariate and multivariable analysis using Cox Proportional Hazards regression with death as the outcome of interest. Time to death or censoring was defined as the time between treatment initiation and outcome date. If the outcome was death, the outcome date was assumed to be the date of death. Patients who were lost to follow-up, or who had their treatment classified as failure, success, or cure were censored at the outcome date. Patients with a follow-up time beyond 9 months were censored at 9 months regardless of the eventual outcome.

A composite variable was created to describe disease type as smear positive pulmonary TB, smear negative pulmonary TB, pulmonary TB without a smear result, and extra-pulmonary TB. To describe HIV and ART status, we evaluated two composite variables. The first classified patients as HIV-negative, HIV-positive and receiving ART, HIV-positive and not receiving ART, and HIV status unknown. The second further categorized patients who were HIV-positive and receiving ART based on the time of ART initiation relative to TB treatment; patients could either have initiated ART before initiating TB treatment, after TB treatment, or at an unknown date.

We evaluated the proportional hazards assumption by testing the significance of time-dependent interaction terms. We included in a multivariable model all variables with evidence of bivariate association (*P* value less than 0.2) and used backward elimination to construct the final model. During multivariable analysis, variables with *P* values greater than 0.05 were eliminated from the model. We used robust standard errors to take into account the potential effect of clustering by health facility. We assessed both a main effects multivariable model and a model including significant two-way interaction terms between covariates. Data was analyzed using Stata version 12.0 and SAS version 9.3.

### Ethical considerations

This analysis involved the use of de-identified secondary data that was collected as part of routine program monitoring. Approval to use TIBU data was obtained from the National TB Control Program. This study was subjected to Human Research Protection review at the U.S. Centers for Disease Control and Prevention which determined it not to constitute human subjects research.

## Results

### Patient population

During 2013–2014, health facilities in Kenya registered 163,618 TB patients aged 15 years and above. Of these, 1,604 (1.0%) were excluded from the analysis because of out-of-range values (age more than 100 years), missing key information (outcome or date of outcome), or inconsistencies (date of treatment outcome before start date), and 162,014 were included in the analysis. Included and excluded patients had similar distribution by sex, HIV status, ART status and year of registration; excluded records had more patients aged ≥75 years and from faith-based facilities.

Of included patients, 57,408 (35.4%) were HIV-infected, with 50,616 (88.2%) of these reporting ART use. A total of 97,019 (59.9%) patients were HIV negative, 7,052 (4.4%) were not tested, and 535 (0.3%) declined HIV testing. Of the HIV-infected patients on ART, 22,318 (44.1%) had a known date of ART initiation; of these, 10,024 (44.9%) had started their ART before TB diagnosis, and 12,294 (55.1%) after TB diagnosis.

### Mortality among TB patients

**Fig 1:**
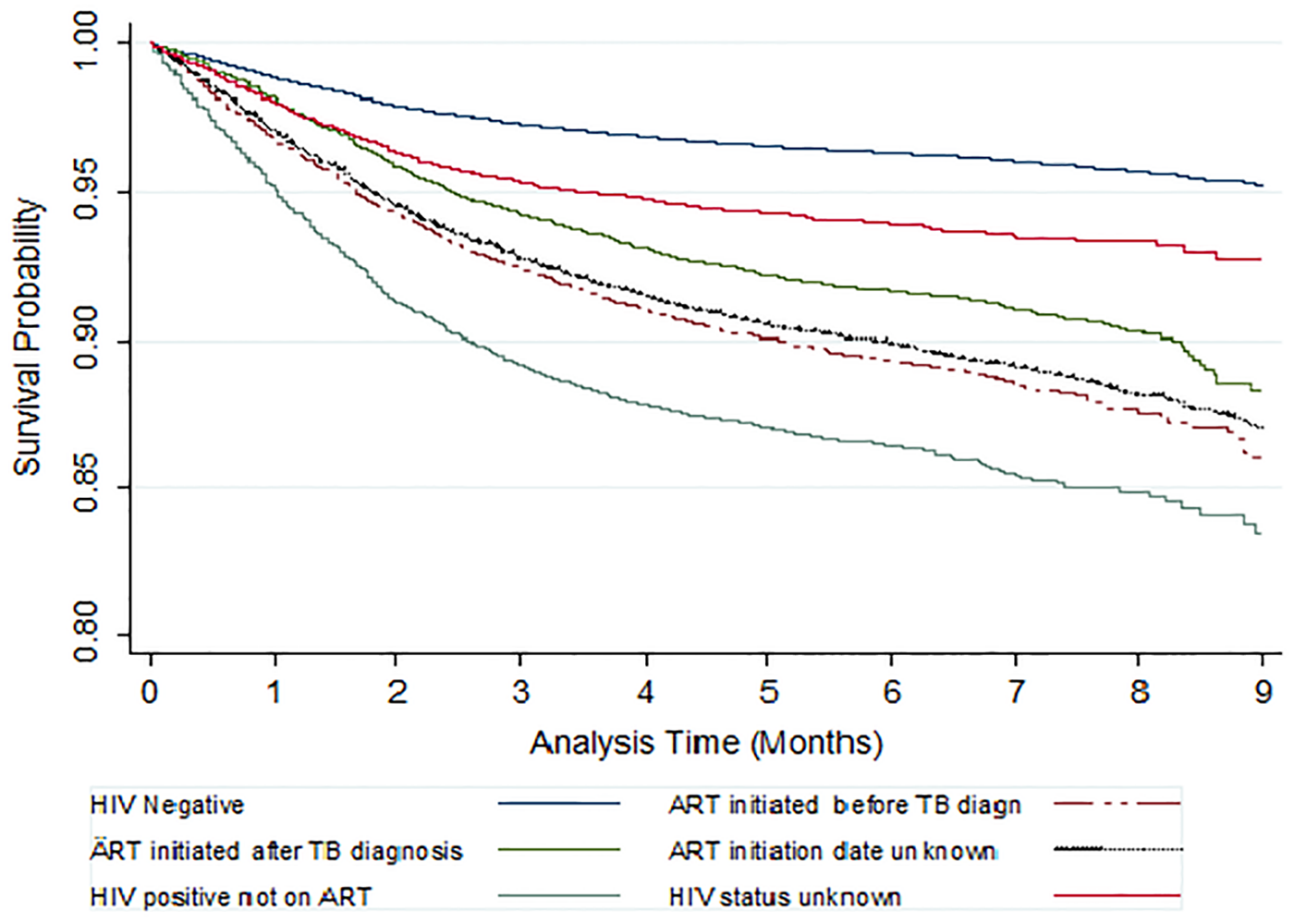
Survival of patients on tuberculosis treatment by their HIV and ART status.

Of the 162,014 patients included in the analysis, 9,907(6.1%) are known to have died; of these, 5,430 (53.6%) died within two months of TB treatment initiation. The case fatality rate was highest among persons with HIV who were not on ART (13.1%) followed by HIV-positive patients on ART (9.9%) and HIV-unknown patients (5.9%), and lowest among HIV-negative patients (3.7%). The crude mortality rate was 12.7 (95% confidence interval CI: 12.5–13.0) per 100 person-years of observation. The probability of survival up to 9 months during treatment was highest among HIV-negative patients (survival probability = 0.95; 95% CI: 0.94–0.95) and lowest among HIV-positive patients who were not on ART (survival probability = 0.83; 95% CI: 0.82–0.85) (Fig 1). The PAF of deaths in TB patients attributable to HIV was 42%.

Compared to the general Kenyan population, TB patients were 10.6 (95% CI: 10.4–10.8) times more likely to die, and the excess mortality rate was 11.5/100 person-years (95% CI: 11.3–11.7) (Table 1). The SMR was similar among men and women and declined with age. Excess mortality was also similar among men and women and somewhat increased with age.

**Table 1:** Excess mortality among TB patients diagnosed in Kenya, 2013 to 2014, by age and sex.

Sex (age group, in years)	Standardized Mortality Rate (95% confidence interval)	Observed mortality rate/100 person-years	Expected mortality rate/100 person-years	Excess mortality/100-person years (95% confidence interval)
Female (15–24)	25.1 (22.9–27.5)	6.6	0.3	6.3 (5.7–6.9)
Female (25–34)	14.9 (14.0–15.7)	11.8	0.8	11.0 (10.4–11.7)
Female (35–44)	12.0 (11.3–12.8)	15.2	1.3	13.9 (13.1–14.8)
Female (45–54)	16.2 (14.8–17.6)	15.7	1.0	14.7 (13.6–16.0)
Female (55–64)	14.1 (12.6–15.8)	17.3	1.3	16.0 (14.4–17.8)
Female (65–74)	7.6 (6.7–8.6)	23.4	3.0	20.4(17.9–22.9)
Female (≥75)	2.2 (1.9–2.6)	32.7	14.9	17.8 (14.6–21.6)
Female (All)	11.6 (11.2–11.9)	12.8	1.1	11.7 (11.4–12.9)
Male (15–24)	14.3 (12.9–15.9)	4.3	0.3	4.0 (3.6–4.4)
Male (25–34)	15.2 (14.4–16.0)	9.1	0.6	8.5 (8.0–8.9)
Male (35–44)	10.1 (9.6–10.6)	13.2	1.3	11.9 (11.3–12.4)
Male (45–54)	12.7 (12.0–13.5)	17.8	1.4	16.4 (15.5–17.3)
Male (55–64)	13.1 (12.2–14.2)	22.3	1.7	20.6(19.2–22.1)
Male (65–74)	7.9 (7.2–8.6)	30.0	3.8	26.2 (24.1–28.4)
Male (≥75)	2.5 (2.2–2.7)	41.4	16.9	24.5 (21.6–27.7)
Male (All)	9.7 (9.5–9.9)	12.6	1.3	11.3 (11.0–11.6)
**Total**	**10.6 (10.4–10.8)**	**12.7**	**1.2**	**11.5 (11.3–11.7)**

#### Risk factors for death among TB patients

In bivariate analysis, age, TB treatment history, disease type, HIV and ART status, and geographic region were significantly associated with death (Table 2). Patients who were HIV positive but not on ART were almost four times likely to die than HIV negative patients (HR = 3.9, 95% CI: 3.6–4.3). The risk of dying increased with increasing age; those aged ≥75 years were seven times more likely to die than those aged 15 to 24 years (HR = 7.0, 95% CI-6.3–7.9).

**Table 2:** Bivariate and multivariable associations between patient characteristics and death among TB patients in Kenya.

Characteristic	Deaths / Registered cases	Percent who died	Crude Hazard Ratio (95% CI)	Adjusted Hazard Ratio from main effects model (95% CI)
**HIV and ART status**				
HIV-negative	3,577 / 97,019	3.7	Reference	Reference
HIV-positive, on ART	4,993 / 50,616	9.9	2.75 (2.61–2.89)	2.60 (2.46–2.74)
ART before TB treatment	1,081 / 10,024	10.8	3.01 (2.78–3.25)	
ART after TB treatment	1,039 / 12,294	8.5	2.33 (2.14–2.53)	
ART date unknown	2,873 / 28,298	10.2	2.84 (2.68–3.01)	
HIV-positive, not on ART	891 / 6,792	13.1	3.93 (3.60–4.30)	4.23 (3.87–4.62)
Unknown HIV status	446 / 7,587	5.9	1.67 (1.48–1.87)	1.65 (1.48–1.85)
Sex*				
Female	3,932 / 63,331	6.2	Reference	
Male	5,975 / 98,682	6.1	0.97 (0.93–1.01)	
**Age group**				
15–24	810 / 31,328	2.6	Reference	Reference
25–34	2,585 / 52,371	4.9	1.93 (1.79–2.08)	1.44 (1.34–1.55)
35–44	2,591 / 38,574	6.7	2.62 (2.42–2.84)	1.68 (1.55–1.82)
45–54	1,703 / 20,766	8.2	3.22 (2.96–3.51)	2.12 (1.94–2.31)
55–64	994 / 10,245	9.7	3.84 (3.49–4.23)	2.89 (2.62–3.18)
65–74	733 / 5,800	12.6	5.12 (4.61–5.68)	4.51 (4.07–5.00)
> = 75	491 / 2,930	16.8	7.02 (6.26–7.88)	6.57 (5.85–7.37)
**Calendar year of enrollment**				
2013	4,843 / 81,194	6.0	Reference	
2014	5,064 / 80,820	6.3	1.04 (1.00–1.09)	
**Disease type**				
Pulmonary, smear-positive	2831 / 76,426	3.7	Reference	Reference
Pulmonary, smear-negative	4186 / 52,301	8.0	2.24 (2.12–2.37)	1.57 (1.48–1.66)
Pulmonary, smear unknown	640 / 6,219	10.3	3.00 (2.71–3.33)	2.29 (2.07–2.53)
Extra-pulmonary	2250 / 27,068	8.3	2.31 (2.16–2.47)	1.88 (1.76–2.00)
**TB treatment history**^†^				
Retreatment/relapse	1,342 / 16,392	8.2	1.31 (1.23–1.39)	1.24 (1.16–1.32)
New	8,499 / 143,990	5.9	Reference	Reference
**Type of health facility**				
Faith based	114 / 2,509	4.5	0.73 (0.50–1.07)	
Private	1,755 / 30,552	5.7	0.92 (0.83–1.03)	
Public	8,038 / 128,953	6.2	Reference	
**Region**				
Central	1,263 / 17,970	7.0	1.73 (1.50–2.00)	1.87 (1.62–2.16)
Coast	902 / 16,894	5.3	1.29 (1.07–1.56)	1.45 (1.22–1.72)
Eastern	1,263 / 24,109	5.2	1.27 (1.09–1.47)	1.54 (1.33–1.79)
Nairobi	1,072 / 26,140	4.1	Reference	Reference
North Eastern	148 / 4190	3.5	0.86 (0.70–1.06)	1.24 (1.01–1.53)
Nyanza	2,391 / 25,462	9.4	2.32 (2.01–2.68)	1.98 (1.72–2.28)
Rift Valley	1,863 / 35,119	5.3	1.30 (1.12–1.52)	1.39 (1.19–1.62)
Western	1,005 / 12,130	8.3	1.99 (1.70–2.33)	1.83 (1.56–2.14)
**Directly observed therapy**				
Observed by household member	8,430 / 139,992	6.0	Reference	
Observed by community volunteer	122 / 1,712	7.1	1.19 (0.95–1.48)	
Observed by healthcare worker	1,347 / 20,168	6.7	1.06 (0.98–1.14)	
Not done	8 / 142	5.6	1.01 (0.50–2.04)	

* Sex violated the proportional hazards assumption, so crude odds ratios are presented. Multivariable analysis is stratified by sex.

^†^1,632 transfer-in patients excluded from analysis.

CI = Confidence interval.

In multivariable analysis, HIV status, age, disease type, treatment history, HIV, and geographic region were independently associated with death (Table 2). In an initial main effects model, compared to patients without HIV, the risk of death among patients with HIV who were receiving ART did not differ substantially for patients who initiated ART before TB treatment (aHR = 2.8, 95% CI: 2.6–3.0) compared to patients who initiated ART after TB treatment (aHR = 2.3, 95% CI: 2.1–2.5). Therefore, for ease of interpretation, we present the multivariable model including the simpler composite variable that included only whether or not the person received ART at any time.

In a main effects model, patients with HIV who were not receiving ART had an over four-fold risk of death compared to patients without HIV (aHR = 4.2, 95% CI: 3.9–4.6). In contrast, patients with HIV who were receiving ART had only 2.6 times the risk of death (aHR = 2.6, 95% CI: 2.5–2.7). Compared to patients without a history of prior treatment, retreatment patients (relapses, returnees after default, and treatment failures) had an increased likelihood of death (aHR = 1.2, 95% CI: 1.2–1.3). In addition, patients from all other regions of Kenya had higher risks of death than patients in Nairobi, with patients from the former Nyanza province having a doubled risk (aHR = 2.0 95% CI: 1.7–2.3).

Significant interactions were observed between HIV status and age, and between HIV status and disease type. A model including these pairwise interaction terms showed that the increased risk of death associated with HIV was more pronounced among younger patients than older patients (Table 3). For HIV-negative patients, the risk of death increased steadily with age, but for patients with HIV, this difference was much less pronounced (S1 Table). Patients who were HIV-positive had elevated mortality regardless of disease type, but the increased risk of death associated with HIV was greater for patients with pulmonary smear-positive disease than other disease types (S2 Table). Patients with all other disease types were more likely to die than patients with pulmonary smear-positive disease, regardless of HIV status (S3 Table). Inclusion of interaction terms in the multivariable model did not affect the association between death and other covariates.

**Table 3:** Adjusted hazard ratios for the association between HIV/ART status and death, stratified by age group, among patients with smear-positive pulmonary disease.

HIV Status	Age Group in Years						
	15–24	25–34	35–44	45–54	55–64	65–74	≥75
**HIV-negative**	reference	reference	reference	reference	reference	reference	reference
**HIV-positive, on ART**	7.85 (6.65–9.27)	5.12 (4.56–5.76)	3.39 (3.00–3.82)	2.74 (2.41–3.12)	2.24 (1.89–2.65)	2.30 (1.86–2.86)	1.47 (0.97–2.23)
**HIV-positive not on ART**	10.25 (7.54–13.92)	8.22 (6.90–9.81)	6.20 (5.13–7.49)	4.79 (3.86–5.95)	3.83 (2.80–5.23)	4.52 (2.87–7.13)	3.08 (1.32–7.19)
**HIV status unknown**	1.97 (1.35–2.89)	2.34 (1.8–3.05)	1.97 (1.5–2.59)	1.55 (1.12–2.14)	2.04 (1.49–2.80)	1.89 (1.35–2.65)	1.64 (1.11–2.40)

Hazard ratios adjusted for TB treatment history and region, and analysis stratified by sex

## Discussion

In this study TB patients experienced a tenfold increase in mortality rates compared with the general population, with 42% of these deaths attributable to HIV. HIV increased the risk of death among TB patients, especially for co-infected patients not receiving ART.

This SMR of 10 that we report is high compared with those reported in previous studies: 8 in Western Kenya [21], 8 in the Netherlands [28], 6 in Spain [29], 4 to 6 in India [15, 16], and 5 in China [30]. The major explanation for this observation may be Kenya’s high HIV prevalence since the burden of TB/HIV co-infected patients is high, and these patients are at a particularly high risk for death. In Kenya, the re-emergence of TB has been linked to the HIV epidemic, and a third of TB cases have been attributed to HIV [5]. The Nyanza region in Western Kenya, which had the highest risk of death among TB patients, also has the highest HIV prevalence.

Excess mortality among TB patients with HIV was expected, even among those on ART [31, 32]. However, mortality was substantially lower among patients on ART compared to those who were HIV-infected but not on ART. The fact that 42% of the deaths in TB patients were attributed to HIV even though 88% of TB/HIV patients received ART suggests that without such high ART coverage, the HIV-associated mortality among TB patients would likely have been higher. Our observation that mortality was similar among those who initiated ART before TB diagnosis and those initiated ART after TB diagnosis could be due to selection bias in ART use. Previously, only HIV-infected people with advanced (based on clinical criteria or low CD4 count) disease were started on ART. The protective effect of ART that was started before diagnosis of TB was probably underestimated because these patients already had an increased likelihood of death due to their advanced immunodeficiency.

Together, these results suggest that TB is associated with a substantial burden of mortality in Kenya and HIV contributes greatly to this risk, and that while ART is protective against death, its protective effect is far from complete. Thus, averting deaths caused by TB will require reducing overall TB risk by improving TB control, reducing risk among people living with HIV and reducing mortality by early initiation of ART among people living with HIV. Overall TB prevention involves broad interventions like intensified case finding, treating all cases (especially smear positive cases), and treatment of latent TB infection. Furthermore, isoniazid preventive therapy (IPT) is effective in preventing TB in the immunocompromised, especially those infected with HIV [33, 34]. Although ART does not eliminate the occurrence of TB in HIV infected people, it reduces the risk of TB by 80% to 92% [35–38].

In addition to preventing TB, ART and IPT have both been proven to reduce mortality. A meta-analysis that included 21 studies (20 routine surveillance data and 1 clinical trial) reported a 44–71% reduction in mortality among TB/HIV co-infected individuals as a result of ART [39]. An econometric analysis of 41 high TB/HIV countries estimated that a 1% increase in ART coverage can result in 27% less TB deaths in the population [40], while a study in USA reported that immediate initiation of ART would reduce mortality by 17% [41]. Additionally, a combination of IPT and ART for persons living with HIV has been shown to reduce the risk of death by at least 60% [42, 43]. Prior to 2016 ART initiation in Kenya followed WHO guidelines which recommended treatment of those with advanced HIV. In 2015, WHO revised ART guidelines to recommend immediate initiation of ART upon HIV diagnosis, commonly referred to as “test and start” [44]. Kenya adopted this guideline in mid 2016 and is currently rolling out the strategy. Our results suggest that rolling out the “test and start” strategy may not only improve general survival and reduce TB incidence, but may also further reduce mortality in HIV-infected TB patients.

We found that retreatment patients had a higher risk of death than new patients. A similar finding has been reported by other studies [14, 15], but contradicts some earlier studies which reported new cases to be at increased risk [20, 45]. One reason why being on a retreatment regimen may be associated with increased mortality is that retreatment patients are more likely to have drug-resistant TB [46, 47]. A study done in Nairobi reported that 17.6% of retreatment cases had resistance to isoniazid and 6.6% had multi drug resistance compared to none among new patients [48]. In Kenya, there is a policy to subject retreatment cases to drug sensitivity testing. In this dataset, however, there was very limited information on drug resistance patterns, so we could not determine the contribution of drug resistance to mortality.

The observation that patients with pulmonary smear-negative disease and extra-pulmonary disease had elevated risks of death could be explained by the nature of these diagnoses. The diagnosis of both smear-negative and extra-pulmonary disease is clinical. Quite often, smear-negative disease is a diagnosis of exclusion. It is therefore possible that increased mortality among smear-negative and extra-pulmonary cases may result from a misdiagnosis, with the result that the true cause of illness such as lung cancer or lymphoma was not treated. For patients who genuinely have TB that is sputum smear-negative, there is a possibility of delayed initiation of anti-TB treatment occasioned by difficulties in making a timely diagnosis [49], especially among HIV infected individuals. The accuracy of clinical examination and diagnostic tests like radiology and sputum smear is lower among HIV infected people due to atypical disease presentation [50]. Timely diagnosis of sputum smear-negative disease can be promoted through utilization of bacteriological confirmation methods such as the Gene Xpert MTB Rif Assay [51], the use of which is currently being expanded in Kenya.

Mortality did not differ by the type of health facility providing treatment. Our finding suggests that TB patients in Kenya get similar standard of care regardless of the type of health facility attended. This is reasonable given that all TB treatment in Kenya is overseen by the national TB program, even when it occurs in private facilities. The finding that the method of observing therapy is not associated with mortality suggests that all of the methods currently used to ensure observation in Kenya are equally effective. Almost all TB patients in Kenya get some form of DOT. TB patients are first registered and counseled at the health facility, then given a choice of having DOT is to be administered at the facility, or at home by a household member or a community health volunteer.

The excess mortality among patients on treatment for TB observed in this study indicates that TB disease increases a person’s risk of mortality regardless of their HIV or ART status. Our data demonstrate that we are still far from achieving the “End TB Strategy” target to end TB deaths globally by 2035 [52]. Consequently, there is an urgent need to redouble efforts to prevent TB by implementing the following strategies which are recommended by WHO: intensified case finding, isoniazid preventive therapy (IPT), infection control and early ART initiation [53]. Aggressive implementation of the above strategies is likely to contribute to reduced TB associated mortality in Kenya.

This study used routine data, which suffers from limitations of missing information and occasionally inconsistent data. Consequently, a small fraction of patients had to be excluded from the analysis. However, it is important to note that the excluded observations were similar to included ones in key variables. Importantly, the national-level data used in this analysis has been validated by other evaluations and determined to be adequate in completeness and accuracy.

In conclusion, mortality among TB patients could be at least ten times higher than in the general population. HIV is a significant contributor to TB-associated mortality, especially among HIV-infected patients who are not on ART. There is urgent need to redouble efforts to prevent TB especially among high risk populations. Early initiation of ART among HIV infected people (test and treat approach) should further reduce TB-associated deaths by preventing TB and reducing mortality among those with TB. Intensified TB case finding among HIV infected people will ensure timely identification of TB/HIV co-infected and timely initiation of TB treatment could further reduce mortality.

## Supporting information

S1 Table Adjusted hazard ratios for the association between age group and death, stratified by HIV/ART status. (DOCX)

S2 Table Adjusted hazard ratios for the association between HIV/ART status and death, stratified by age group and disease type. (DOCX)

S3 Table Adjusted hazard ratios for the association between disease type and death, stratified by HIV/ART status. (DOCX)

S4 Table CDC Approval_Kenya Mortality: Non-human subjects research determination. (PDF)
