# Supplementary material for: Reduction of HIV-associated excess mortality by antiretroviral treatment among tuberculosis patients in Kenya
Source: PLoS One. 2017 Nov 16;12(11):e0188235. doi: 10.1371/journal.pone.0188235 (PMC5690617; doi:10.1371/journal.pone.0188235)
Supplement: S1 Table — (DOCX) [file pone.0188235.s001.docx]

S1 Table: Adjusted hazard ratios for the association between age group and death, stratified by HIV/ART status

| **Age group** | **HIV status** | | | |
| --- | --- | --- | --- | --- |
|  | **HIV-negative** | **HIV-positive,  on ART** | **HIV-positive, not on ART** | **HIV status unknown** |
| **15-24** | Reference | Reference | Reference | Reference |
| **25-34** | 1.60 (1.40–1.82) | 1.04 (0.94–1.16) | 1.28 (0.97–1.69) | 1.90 (1.30–2.77) |
| **35-44** | 2.46 (2.14–2.82) | 1.06 (0.95–1.18) | 1.49 (1.12–1.98) | 2.46 (1.66–3.63) |
| **45-54** | 3.60 (3.13–4.14) | 1.26 (1.12–1.41) | 1.68 (1.23–2.29) | 2.83 (1.84–4.36) |
| **55-64** | 5.02 (4.36–5.79) | 1.43 (1.24–1.66) | 1.88 (1.30–2.72) | 5.20 (3.48–7.78) |
| **65-74** | 6.90 (5.97–7.99) | 2.03 (1.65–2.48) | 3.05 (1.84–5.05) | 6.62 (4.35–10.07) |
| **≥75** | 9.85 (8.48–11.45) | 1.85 (1.24–2.77) | 2.96 (1.23–7.12) | 8.18 (5.24–12.76) |

Hazard ratios adjusted for TB treatment history and region, and analysis stratified by sex.
