# Supplementary material for: Reduction of HIV-associated excess mortality by antiretroviral treatment among tuberculosis patients in Kenya
Source: PLoS One. 2017 Nov 16;12(11):e0188235. doi: 10.1371/journal.pone.0188235 (PMC5690617; doi:10.1371/journal.pone.0188235)
Supplement: S2 Table — (DOCX) [file pone.0188235.s002.docx]

S2 Table: Adjusted hazard ratios for the association between HIV/ART status and death, stratified by age group and disease type

| **HIV Status** | **Type of TB** | | | | |
| --- | --- | --- | --- | --- | --- |
|  | **Age group** | **Pulmonary,  smear-positive** | **Pulmonary, smear-negative** | **Pulmonary, smear unknown** | **Extra-pulmonary** |
| **HIV-positive on ART, compared to HIV-negative** | **15-24** | 7.85 (6.65–9.27) | 5.14 (4.36–6.06) | 5.53 (4.33–7.05) | 4.51 (3.80–5.35) |
|  | **25-34** | 5.12 (4.56–5.76) | 3.35 (2.99–3.76) | 3.61 (2.93–4.45) | 2.94 (2.61–3.32) |
|  | **35-44** | 3.39 (3.00–3.82) | 2.22 (1.98–2.49) | 2.39 (1.94–2.93) | 1.95 (1.71–2.22) |
|  | **45-54** | 2.74 (2.41–3.12) | 1.79 (1.59–2.03) | 1.93 (1.56–2.40) | 1.57 (1.38–1.8) |
|  | **55-64** | 2.24 (1.89–2.65) | 1.47 (1.26–1.71) | 1.58 (1.25–1.99) | 1.29 (1.09–1.52) |
|  | **65-74** | 2.30 (1.86–2.86) | 1.51 (1.23–1.84) | 1.62 (1.24–2.13) | 1.32 (1.07–1.64) |
|  | **≥75** | 1.47 (0.97–2.23) | 0.96 (0.64–1.45) | 1.04 (0.67–1.61) | 0.85 (0.56–1.28) |
| **HIV-positive not on ART, compared to HIV-negative** | **15-24** | 10.25 (7.54–13.92) | 6.49 (4.78–8.79) | 6.52 (4.47–9.52) | 4.98 (3.58–6.94) |
|  | **25-34** | 8.22 (6.90–9.81) | 5.20 (4.34–6.24) | 5.24 (3.88–7.06) | 4.00 (3.23–4.95) |
|  | **35-44** | 6.2 0(5.13–7.49) | 3.92 (3.27–4.70) | 3.95 (2.95–5.28) | 3.01 (2.44–3.72) |
|  | **45-54** | 4.79 (3.86–5.95) | 3.03 (2.46–3.73) | 3.05 (2.24–4.15) | 2.33 (1.84–2.95) |
|  | **55-64** | 3.83 (2.80–5.23) | 2.42 (1.78–3.30) | 2.44 (1.66–3.58) | 1.86 (1.35–2.56) |
|  | **65-74** | 4.52 (2.87–7.13) | 2.86 (1.85–4.43) | 2.88 (1.79–4.62) | 2.20 (1.37–3.51) |
|  | **≥75** | 3.08 (1.32–7.19) | 1.95 (0.82–4.60) | 1.96 (0.81–4.75) | 1.50 (0.65–3.45) |
| **HIV status unknown, compared to HIV-negative** | **15-24** | 1.97 (1.35–2.89) | 1.65 (1.13–2.43) | 1.46 (0.91–2.32) | 1.46 (0.99–2.17) |
|  | **25-34** | 2.34 (1.80–3.05) | 1.97 (1.49–2.60) | 1.73 (1.18–2.54) | 1.74 (1.30–2.33) |
|  | **35-44** | 1.97 (1.50–2.59) | 1.65 (1.25–2.20) | 1.46 (0.97–2.17) | 1.46 (1.09–1.96) |
|  | **45-54** | 1.55 (1.12–2.14) | 1.30 (0.95–1.77) | 1.14 (0.75–1.74) | 1.15 (0.84–1.58) |
|  | **55-64** | 2.04 (1.49–2.80) | 1.71 (1.28–2.29) | 1.51 (1.01–2.26) | 1.52 (1.12–2.05) |
|  | **65-74** | 1.89 (1.35–2.65) | 1.59 (1.19–2.12) | 1.40 (0.92–2.11) | 1.40 (1.02–1.93) |
|  | **≥75** | 1.64 (1.11–2.40) | 1.37 (0.98–1.92) | 1.21 (0.77–1.89) | 1.21 (0.84–1.75) |

Hazard ratios adjusted for TB treatment history and region, and analysis stratified by sex
