# Supplementary material for: Reduction of HIV-associated excess mortality by antiretroviral treatment among tuberculosis patients in Kenya
Source: PLoS One. 2017 Nov 16;12(11):e0188235. doi: 10.1371/journal.pone.0188235 (PMC5690617; doi:10.1371/journal.pone.0188235)
Supplement: S3 Table — (DOCX) [file pone.0188235.s003.docx]

S3 Table: Adjusted hazard ratios for the association between disease type and death, stratified by HIV/ART status

| **Type of TB** | **HIV status** | | | |
| --- | --- | --- | --- | --- |
|  | **HIV-negative** | **HIV-positive,  on ART** | **HIV-positive, not on ART** | **HIV status unknown** |
| **Pulmonary,  smear-positive** | Reference | Reference | Reference | Reference |
| **Pulmonary,  smear-negative** | 1.99 (1.81–2.19) | 1.30 (1.21–1.4) | 1.26 (1.06–1.49) | 1.67 (1.30–2.14) |
| **Pulmonary,  smear result unknown** | 2.84 (2.41–3.35) | 2.00 (1.74–2.29) | 1.81 (1.37–2.37) | 2.10 (1.50–2.94) |
| **Extra-pulmonary** | 2.62 (2.37–2.88) | 1.50 (1.38–1.63) | 1.27 (1.04–1.55) | 1.94 (1.49–2.54) |

Hazard ratios adjusted for TB treatment history and region, and analysis stratified by sex
