# Supplementary material for: Reduction of HIV-associated excess mortality by antiretroviral treatment among tuberculosis patients in Kenya
Source: PLoS One. 2017 Nov 16;12(11):e0188235. doi: 10.1371/journal.pone.0188235 (PMC5690617; doi:10.1371/journal.pone.0188235)
Supplement: S4 Table — (PDF) [file pone.0188235.s004.pdf]

CGH HSR Tracking #: 2016-322

Request for Project Determination & Approval – Center for Global Health (CGH)

This form should be used to submit proposals to the CGH Office of the Associate Director for Science/Laboratory Science (ADS/ADLS) for research/nonresearch determination and requirements for IRB review/approval.
Approval Chain: Investigator → Branch Chief/Country Director →Division ADS → CGH Human Subjects Mailbox

☑ **New Request** ☐ **Amendment** ☐ **Laboratory Submission**

Project Title: Excess Mortality and Risk Factors Associated with Mortality among Tuberculosis Patients in Kenya		Project Location/Country(ies): Kenya
CDC Principal Investigator (SEV#): Martinus Borgdorf (SEV 2423)		
CDC Project Officer(SEV#): Martinus Borgdorff (SEV 2423)	Division: OD	Telephone: 254722772609
Proposed Start Date (mm/dd/yyyy): 10/15/2016	Proposed End Date (mm/dd/yyyy):	

Collaborating Institutions (List other collaborating institutions in the protocol or in a separate document)

☐CoAg ☐Grant ☐contract #		IRB Exp. Date (if applicable):
Title of CoAg, Grant, or Contract		
Supported Institution/Entity Name	MINISTRY OF HEALTH	
Supported Institution/Entity FWA#	FWA00006828	FWA Exp. Date (mm/dd/yyyy): 06/15/2020

Please check appropriate category and subcategory:

☑ **I. Activity is NOT human subjects research. Primary intent is public health practice or a disease control activity (Check all that apply)**

- ☐ **A.** Epidemic or endemic disease control activity; if applicable, Epi-AID #
- ☑ **B.** Routine surveillance activity (e.g., disease, adverse events, injuries)
- ☐ **C.** Program evaluation activity*
- ☐ **D.** Public health program activity$^{\Omega}$
- ☐ **E.** Laboratory proficiency testing

* Evaluation of a new intervention for effectiveness and comparison of different interventions are research under CDC policy.
Ω e.g., service delivery; health education programs; social marketing campaigns; program monitoring; electronic database construction and/or support; development of patient registries; needs assessments; and demonstration projects intended to assess organizational needs, management, and human resource requirements for implementation.

☐ **II. Activity is research but does NOT involve human subjects (Check all that apply)**

- ☐ **A.** Activity is research involving collection or analysis of data about health facilities or other organizations or units (NOT persons).
- ☐ **B.** Activity is research involving data or specimens from deceased persons.
- ☐ **C.** Activity is research involving unlinked or anonymous data or specimens collected for another purpose.
- ☐ **D.** Activity is research involving data or specimens from animal subjects. §

§Note: Approval by CDC Institutional Animal Care and Use Committee (IACUC) may be required for certain animal research. Institution must also have assurance with the Office of Laboratory and Animal Welfare at NIH.

☐ **III. Activity is research involving human subjects but CDC involvement does not constitute "engagement in human subject research."** CDC employees or agents will not intervene or interact with living individuals or have access to identifiable information for research purposes. Appropriate IRB or ethics committee approval is required prior to approval. **(Check all that apply)**

- ☐ **A.** This project is funded under a grant/cooperative agreement/contract award mechanism.
- ☐ **B.** CDC staff provide technical support that does not involve possession or analysis of identifiable data or interaction with participants from whom data are being collected (No CDC Support$^{\beta}$).
- ☐ **C.** CDC staff are involved only in manuscript writing for a project that has closed. For the project, CDC staff did not interact with participants and were not involved with data collection (No CDC Support).
- ☐ **D.** Activity is research involving linked data, but CDC non-disclosure form 0.1375B is signed.$^{\infty}$

β See definition of support on page 3.
∞ Access to linked data is permitted under any of the above sub-categories if CDC investigators and the holder of the key linking the data to identifiable human subjects enter into an agreement using CDC form 0.1375B, prohibiting the release of the key to CDC investigators under any circumstances. The purposes of the planned research do not contradict the terms of consent under which the information or specimens were collected, whether that consent was documented or not documented.

☐ **IV. Activity is research involving human subjects that requires submission to CDC Human Research Protection Office (Check one)** $^{\alpha}$

- ☐ A. Full Board Review (Use forms 0.1250, 0.1370-research partners)
- ☐ B. Expedited Review (Use same forms as A above)
- ☐ C. Exemption Request** (Use forms 0.1250X, 0.1370-research partners)
- ☐ D. Reliance¥
  - ☐ 1. Request to allow CDC to rely on a non-CDC IRB (Use same forms as A above, plus 0.1371)
  - ☐ 2. Request to allow outside institution to rely on CDC IRB (Use same forms as A above, plus 0.1372)

α There are other types of requests not listed under category IV, e.g., continuation of existing protocol, amendment, incident reports.

¥ Exemption and reliance request is approved by CDC Human Research Protection Office (HRPO).

CGH HSR Tracking #: 2016-322

Amendment: If this request is an amendment to an existing project determination. Please include a brief description of the substantive change or modification below and attach both clean and marked copies of the amended protocol or project outline.

Submission: Attach a protocol or project description (See standard format below) in enough detail to justify the proposed category. Submit your request to your branch chief (or country director for DGHA country staff).

Approval Chain
Investigator → **Branch Chief/Country Director →Division ADS → CGH Human Subjects Mailbox**

CGH ADS/ADLS Review **Date received in CGH ADS /ADLS office:**

[X] **Project does not require human subject research review beyond CGH at this time.**

[ ] **Project constitutes human subject research that must be routed to CDC HRPO.**

Comments/Rationale for Determination:

This analysis will benefit the National Tuberculosis Control Program.

**Approvals/Signatures:**	**Date:**	**Remarks:**
Investigator [signature]	10/7/16	
Branch Chief/Country Director [signature]	10/7/16	
[signature], ADS, DGHT<br>Division Human Research Protection Coordinator<br>Division ADS/ADLS or Director	11/28/16	
Natalie Brown<br>CGH Human Research Protection Coordinator<br>CGH ADS/ADLS or Deputy ADS/ADLS	12/2/2016	

Note: Although CDC IRB review is not required for certain projects (categories I,II & III) approved under this determination, CDC investigators and project officers are expected to adhere to the highest ethical standards of conduct and to respect and protect to the extent possible the privacy, confidentiality, and autonomy of participants. All applicable country, state, and federal laws must be followed. Informed consent may be appropriate and should address all applicable elements of informed consent. CDC investigators should incorporate diverse perspectives that respect the values, beliefs, and cultures of the people in the country, state, and community in which they work.
